# Association of Systemic Inflammation and Malnutrition With Survival in Nasopharyngeal Carcinoma Undergoing Chemoradiotherapy: Results From a Multicenter Cohort Study

**DOI:** 10.3389/fonc.2021.766398

**Published:** 2021-10-26

**Authors:** Xin Wang, Ming Yang, Yizhong Ge, Meng Tang, Benqiang Rao, Yongbing Chen, Hongxia Xu, Minghua Cong, Zengqing Guo, Hanping Shi

**Affiliations:** ^1^Departments of Gastrointestinal Surgery and Clinical Nutrition, Beijing Shijitan Hospital, Beijing, China; ^2^Department of Oncology, Capital Medical University/Ninth Clinical Medical College, Peking University, Beijing, China; ^3^Department of Clinical Nutrition, Daping Hospital, Army Medical University, Chongqing, China; ^4^Comprehensive Oncology Department, National Cancer Center/Cancer Hospital, Chinese Academy of Medical Sciences and Peking Union Medical College, Beijing, China; ^5^Department of Medical Oncology, Fujian Cancer Hospital, Fujian Medical University Cancer Hospital, Fuzhou, China

**Keywords:** nasopharyngeal carcinoma, PG-SGA, neutrophil-to-lymphocyte ratio (NLR), malnutrition, systemic inflammation, chemoradiotherapy

## Abstract

**Background:**

Malnutrition and systemic inflammation are common in patients with nasopharyngeal carcinoma (NPC). The Patient-Generated Subjective Global Assessment (PG-SGA) score and neutrophil-to-lymphocyte ratio (NLR) reflect the integrated nutritional status and inflammatory level of patients with NPC, respectively. We performed this study to identify whether NLR and PG-SGA score are associated with outcome and survival time for patients with NPC undergoing chemoradiotherapy.

**Methods:**

The multicenter cohort study included 1,102 patients with NPC between June 2012 and December 2019. The associations of all-cause mortality with NLR and PG-SGA score were calculated using the Kaplan–Meier method and the log-rank test. We also did a multivariate-adjusted Cox regression analysis to identify the independent significance of different parameters. Restricted cubic spline regression was carried out to evaluate the association between NLR and overall survival (OS). A nomogram was established using the independent prognostic variables. Interaction terms were used to investigate whether there was an interactive association between NLR and PG-SGA.

**Results:**

A total of 923 patients with NPC undergoing chemoradiotherapy were included in this study: 672 (72.8%) were males and 251 (27.2%) were females, with a mean age of 49.3 ± 11.5 years. The Kaplan–Meier curves revealed that patients with malnutrition (PG-SGA score >3) had worse survival than patients who were in the well-nourished group (PG-SGA score ≤3) (p < 0.0001). In addition, patients in the high NLR group (NLR ≥ 3) had worse survival than those in the low NLR group (NLR < 3) (p < 0.0001). Patients with high PG-SGA and high NLR had the worst survival (p < 0.0001). An increase in NLR had an inverted L-shaped dose–response association with all-cause mortality. A nomogram was developed by incorporating domains of NLR and PG-SGA score to accurately predict OS 12–60 months for patients [the C-index for OS prediction of nomogram was 0.75 (95% CI, 0.70–0.80)]. The interaction of PG-SGA with NLR was significant (p = 0.009). Patients with high PG-SGA and high NLR had a nearly 4.5-fold increased risk of death (HR = 4.43, 95% CI = 2.60–7.56) as compared with patients with low PG-SGA and low NLR.

**Conclusions:**

Our study provided clear evidence that high PG-SGA score and high NLR adversely and interactively affects the OS of patients with NPC undergoing chemoradiotherapy.

## Introduction

Nasopharyngeal carcinoma (NPC) is a specific head and neck epithelial malignant tumor with obvious endemic and racial distribution differences, especially in southern China, with the incidence ranging from 20 to 30 per 100,000 (1).

Due to the complex anatomical location, high sensitivity of irradiation, and a locoregional advanced presentation at diagnosis, the primary treatment for non-disseminated NPC is not radical surgery but radiotherapy (RT) alone or concurrent chemoradiotherapy (CCRT), which is recognized as the most effective and reasonable strategy to control local recurrence and prolong survival time (2, 3). However, the therapeutic effect is unsatisfactory (4). More than 20% of the patients with advanced disease still develop local recurrence and distant metastases even after radical RT and systemic chemotherapy (5). Recurrent or metastatic NPC is associated with poor prognosis, with a median survival period of approximately 17 months (6). Therefore, improving the treatment effect and survival time of NPC patients remains challenging.

In clinical practice, malnutrition is one of major problems that can affect the curative effect and prognosis for people with NPC; 35% of NPC patients lose more than 5% of their weight (7). Even for newly diagnosed patients with NPC, through ideal weight percentage and serum albumin level to evaluate the nutritional status, malnutrition has been estimated in 36.5% and 34.6%, respectively (8). RT will cause further aggravation of malnutrition *via* mucositis, reaching a peak in 3 weeks. A clinical observational study reported that 20.19% of patients with NPC under RT were able to lose 10% of their body weight within 2 months (9). Furthermore, side effect, fatigue, anorexia, etc. often lead to malnutrition during the process of chemotherapy for NPC (10).

Malnutrition and weight loss may impair immune function, restrict vitality, reduce resistance to the disease, and decrease wound healing, which lead to complications, treatment-related toxicities, and resistance to cancer treatment (11). In addition, the effectiveness of the chemotherapy and RT will be significantly reduced, if nutritional status of patients becomes so bad (12). The treatments had to be suspended even and terminated early because of intolerance. Therefore, it is not surprising that malnutrition or weight loss during treatments has been found to be significantly associated with poorer survival outcomes and impaired the quality of life (QoL) in NPC. Furthermore, an increase in 20% weight reduction over the course of treatments was significantly associated with toxicity and mortality during RT in NPC (13).

The Patient-Generated Subjective Global Assessment (PG-SGA) was proposed in 1996 and developed by the European Society for Clinical Nutrition and Metabolism (ESPEN), which is a tool that enables evaluation of the nutritional status for patients with cancer (14). This scoring system has two main parts: one questionnaire for the patient with four questions and one questionnaire for the physician. The questionnaire for the patient includes weight change, symptoms, daily diet, and daily activities; the questionnaire for the physician includes diagnosis, physical examinations, metabolic evaluation, and nutrition-related complications. Finally, the grades for nutritional assessment are divided into the following categories: good nutritional status, suspicious malnutrition, moderate malnutrition, and severe malnutrition. A patient with over 3 points would be regarded as relatively malnourished (15). Thus, PG-SGA has been used for guiding nutritional treatment, making clinical decisions, and predicting outcomes in various cancers, including NPC (16).

In recent years, an increasing number of studies have focused on the influence of nutrition and systemic inflammation on the prognosis of patients with cancer (17, 18). Systemic inflammatory response has been shown to be closely associated with weight loss and malnutrition in NPC (4, 9). Neutrophil-to-lymphocyte ratio (NLR), which is calculated based on the absolute neutrophil count divided by the absolute lymphocyte count, is known to be an indicator of both the immune system and systemic inflammation of cancer patients. NLR and other systemic inflammation-related biomarkers [such as prognostic nutritional index (PNI) (19), platelet-to-lymphocyte ratio (PLR) (20), C-reactive protein (CRP) (21)] are commonly used to predict clinical outcomes for patients with various malignancies (17, 18).

The clinical predictive value of a combination of PG-SGA score and NLR has rarely been investigated in patients with NPC undergoing chemoradiotherapy. Therefore, we performed this paper to identify which prognostic factors may help select patients who will benefit from more aggressive treatment and to evaluate the relationship between nutritional status and systemic inflammation in patients with NPC undergoing chemoradiotherapy.

## Subjects and Methods

### Study Design and Participants

The retrospective data were collected from a multicenter, prospective, observational cohort study named the Investigation on Nutrition Status and its Clinical Outcome of Common Cancers (INSCOC) project of China (chictr.org.cn, registration number: ChiCTR1800020329). In the present study, a total of 1,102 patients with NPC were recruited from 40 clinical centers throughout the China from June 2012 to December 2019, who had to meet the following inclusion criteria: 1) aged 18 to 80 years; 2) with pathological diagnosis of NPC; 3) received radical RT, chemotherapy, or both; 4) without liver and kidney dysfunction; and 5) obtained complete baseline clinical information and laboratory data and complete follow-up data.

### Data Collection and Variable Definition

Within the first 48 h after hospital admission, demographic and clinicopathological data were collected by trained investigators, including gender, age at diagnosis, body mass index (BMI), smoking status, comorbidities, alcohol consumption, tea consumption, family history of cancer, tumor stage, NLR, and PG-SGA score. The nutritional status of all patients was evaluated using the PG-SGA: a patient with over 3 points would be regarded as relatively malnourished (15); in contrast, less than or equal to 3 points was classified into the well-nourished group. BMI was calculated by body weight in kilograms divided by the height in squared meters (kg/m^2^). NLR was used as a continuous variable, and widely accepted values were used to group patients. The optimal cutoff values (“normal” (<3) and “moderate and high” (≥3) inflammation) for NLR were generated according to reference (17). The tumor stage in all patients was confirmed by pathological examination. NPC patients were staged according to the 8th edition of the American Joint Committee on Cancer/Union for International Cancer Control (AJCC/UICC) TNM staging system. The types of chemotherapy or RT included curative, neoadjuvant, adjuvant, maintenance, and palliative types in the study. All patients were regularly followed up *via* outpatient visits or telephone interviews until death, last contact on March 31, 2020. The study was approved by the research ethical committees of all participating institutions and was designed in accordance with the Declaration of Helsinki, and all participants signed the consent before study entry.

### Statistical Analysis

Categorical variables were expressed as whole numbers and percentages and were compared by the chi‐square test or Fisher’s exact test, as appropriate. Quantitative variables were reported as mean ± standard deviation (SD), and differences were analyzed using Student’s t-test or the Mann–Whitney U test for groups without a normal distribution. Overall survival (OS) was calculated using the Kaplan–Meier method and the log-rank test, and hazard ratios (HRs) together with 95% CIs were calculated using a univariate Cox regression analysis to investigate the association between potential predictors and mortality. We also did a multivariate-adjusted Cox regression analysis using backward selection to identify the independent significance of different parameters. Restricted cubic spline regression was carried out to evaluate the association between NLR and OS. Based on the results of multivariable analysis, we formulated a nomogram using the “rms” package in R to predict the probability of 12-, 24-, 36-, 48-, and 60-month OS rates for NPC patients after chemoradiotherapy. Harrell’s C-index was used to evaluate discernibility ability of the nomogram. The area under the receiver operating characteristic (ROC) curve (AUC) was used to evaluate the predictive accuracy of the 12-, 24-, 36-, and 48-month OS. Interaction terms were used to investigate whether there was an interactive association between NLR and PG-SGA score. All statistical analyses were performed with R software (version 3.6.2, http://www.rproject.org). p < 0.05 from the two-sided test was considered statistically significant.

## Results

### Clinical Characteristics of the Investigated Nasopharyngeal Carcinoma Patients

**Figure 1:**
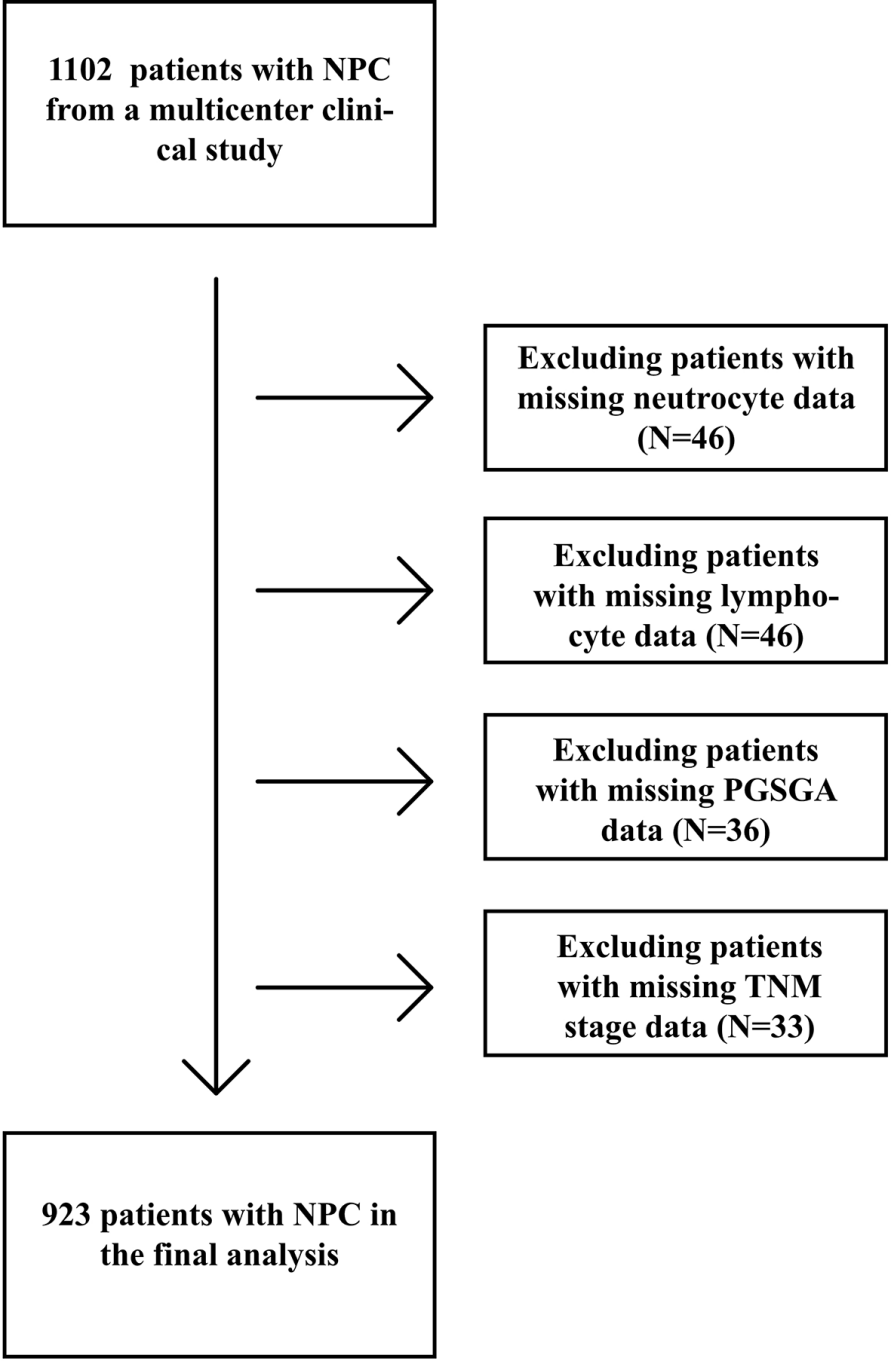
Flowchart of the study design.

A total of 923 patients with NPC undergoing chemoradiotherapy were included in this study; the flowchart of screening process is shown in **Figure 1**. One hundred sixty-one patients were excluded due to missing key data or variables in our analysis. A total of 18 cases were lost to follow-up. The follow-up rate was 98.4%. Among the remaining cases, the average follow-up time was 28.3 ± 16.3 months, during which there were 99 deaths. The general characteristics of all participants with NPC undergoing chemoradiotherapy by category of PG-SGA score are shown in **Table 1**. There were 672 (72.8%) males and 251 (27.2%) females, with a mean age of 49.3 ± 11.5 years. Among all NPC patients, the PG-SGA-diagnosed severe malnutrition rate was 16.4% (151 patients, determined by the PG-SGA score >3). The TNM stage and BMI between patients with PG-SGA score >3 and PG-SGA score ≤3 had significant difference (p < 0.001).

**Table 1:** Demographic and clinical characteristics of patients with NPC undergoing chemoradiotherapy stratified by PG-SGA.

*Characteristics*	*No. of patients with PG-SGA ≤ 8 (n = 772)*	*No. of patients with PG-SGA > 8 (n = 151)*	*p-Value*
Sex^a^ (male)	571 (74.0)	101 (66.9)	0.092
Age in years^b^	49.00 (11.25)	50.77 (12.51)	0.083
BMI^b^ (kg/m^2^)	23.39 (3.21)	21.54 (3.30)	<0.001
Smoking status^a,c^ (yes)	364 (47.2)	79 (52.3)	0.283
*Alcohol consumption^a,d^ (yes)*	179 (23.2)	42 (27.8)	0.265
Tea drinking status^a,e^ (yes)	254 (32.9)	38 (25.2)	0.076
Hypertension^a^ (yes)	87 (11.3)	13 (8.6)	0.413
*Diabetes^a^ (yes)*	36 (4.7)	8 (5.3)	0.900
Coronary heart disease^a^ (yes)	9 (1.2)	1 (0.7)	0.907
Family history of cancer^a^ (yes)	120 (15.5)	17 (11.3)	0.219
TNM stage^a^			<0.001
I, II, III	493 (63.9)	72 (47.7)	
IV	279 (36.1)	79 (52.3)	
NLR	5.35 (1.99)	5.21 (0.93)	0.466

NPC, nasopharyngeal carcinoma; PG-SGA, Patient-Generated Subjective Global Assessment; BMI, body mass index; NLR, neutrophil-to-lymphocyte ratio.

a Categorical variables are presented as number (percentage).

b Continuous variables are presented as mean (standard deviation).

c The standard is to smoke more than 20 cigarettes in a lifetime.

d The standard is regular drinking in the past year.

e The standard is regular drinking tea in the past year.

### Risk Factors of All-Cause Mortality for Patients With Nasopharyngeal Carcinoma Undergoing Chemoradiotherapy Were Determined Using Univariate and Multivariate Cox Regression Analyses

**Figure 2:**
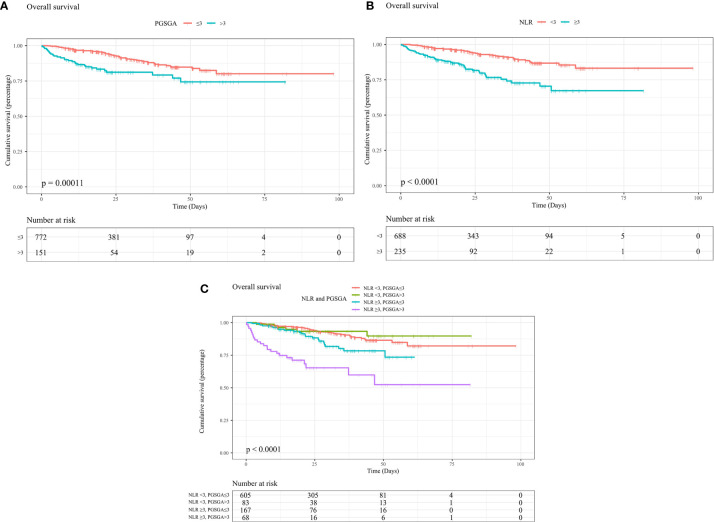
Kaplan–Meier curves show survival rates for patients with NPC undergoing chemoradiotherapy with **(A)** PG-SGA (PG-SGA score >3, ≤3), with **(B)** NLR (NLR ≥ 3, < 3), with **(C)** NLR and PG-SGA (high PG-SGA and high NLR group, high PG-SGA and low NLR group, low PG-SGA and high NLR group, and low PG-SGA and low NLR group). NPC, nasopharyngeal carcinoma; PG-SGA, Patient-Generated Subjective Global Assessment; NLR, neutrophil-to-lymphocyte ratio.

The Kaplan–Meier curves revealed that patients with NPC undergoing chemoradiotherapy, with malnutrition (PG-SGA score >3), had worse survival (HR = 1.441, 95% CI = 0.90–2.31, median OS [MOS] = 21.32 months) than patients with NPC who were in the well-nourished group (PG-SGA score ≤3) (MOS = 24.51 months, p < 0.0001, **Figure 2A**). In addition, patients in the high NLR group (NLR ≥ 3) had worse survival (HR = 2.36, 95% CI = 1.56–3.56, MOS = 21.16 months) than those in the low NLR group (NLR < 3) (MOS = 24.86 months, p < 0.0001, **Figure 2B**). After the study population was further stratified into four subgroups, patients with high PG-SGA (PG-SGA score >3) and high NLR (NLR ≥ 3) had the worst survival (HR = 4.43, 95% CI = 2.60–7.56, p < 0.0001, **Figure 2C**), compared with those in the high PG-SGA and low NLR group, the low PG-SGA and high NLR group, and the low PG-SGA and low NLR group.

We carried out multivariate analyses that included patient age, sex, BMI, alcohol consumption, TNM stage, PG-SGA, and NLR. As shown in **Table 2**, age (HR, 1.040, 95% CI 1.022–1.059; p < 0.001), PG-SGA (HR, 1.070, 95% CI 1.025–1.117; p = 0.002) and NLR (HR, 1.199, 95% CI 1.119–1.285; p < 0.001) were significantly associated with OS. In multivariable Cox proportional hazards models (**Table 3**), high NLR (NLR ≥ 3) was associated with a 135.9% (HR, 2.36; 95% CI, 1.56–3.56) greater risk of death, as compared with those with low NLR (NLR < 3). An increase in 1 score of PG-SGA was associated with a 7% increased risk of death. Additionally, NLR was divided into quartiles; the fourth quartile (≥3) was positively correlated with a worse prognosis (p < 0.001), compared with the first (< 1.61), second (≥1.61, <2.15), and third (≥2.15, <3) quartiles. After the confounding factors were adjusted, HRs of all-cause mortality (HR, 95% CI) were 1.29 (0.63–2.62), 1.38 (0.71–2.70), and 2.90 (1.57–5.34) for the second, third, and fourth quartiles, respectively, showing an increasing trend in the risk of death.

**Table 2:** Association between clinical variables and OS in patients with NPC undergoing chemoradiotherapy.

Variables	Univariate analysis		Multivariate analysis^a^	
	HR (95% CI)	p-Value	HR (95% CI)	p-Value
Sex	0.546 (0.324, 0.921)	0.023	0.715 (0.409, 1.248)	0.238
Age	1.044 (1.025, 1.062)	<0.001	1.040 (1.022, 1.059)	<0.001
BMI	0.903 (0.848, 0.962)	0.002	0.942 (0.882, 1.006)	0.075
Smoking status^b^	1.342 (0.903, 1.993)	0.145		
Alcohol consumption^c^	1.652 (1.083, 2.518)	0.020	1.491 (0.951, 2.338)	0.082
Tea drinking status^d^	1.098 (0.723, 1.667)	0.662		
Hypertension	1.505 (0.855, 2.649)	0.157		
Diabetes	1.624 (0.753, 3.503)	0.216		
Coronary heart disease	0.965 (0.674, 1.381)	0.845		
Family history of cancer	0.686 (0.375, 1.255)	0.221		
TNM stage				
I, II, III	Reference		Reference	
IV	1.914 (1.289, 2.841)	0.001	1.393 (0.920, 2.110)	0.118
PG-SGA	1.098 (1.061, 1.136)	<0.001	1.070 (1.025, 1.117)	0.002
NLR	1.246 (1.172, 1.325)	<0.001	1.199 (1.119, 1.285)	<0.001

NPC, nasopharyngeal carcinoma; OS, overall survival; HR, hazard ratio; CI, confidence interval; BMI, body mass index; PG-SGA, Patient-Generated Subjective Global Assessment; NLR, neutrophil-to-lymphocyte ratio.

a The variables that showed prognostic role in univariate analysis or considered clinically significant were involved in multivariate analysis.

b The standard is to smoke more than 20 cigarettes in a lifetime.

c The standard is regular drinking in the past year.

d The standard is regular drinking tea in the past year.

**Table 3:** Association between PG-SGA or NLR and OS in patients with NPC undergoing chemoradiotherapy.

Variables	Patients (n)	Crude HR^c^ (95% CI)	p-Value	Adjusted HR (95% CI)	p-Value
**PG-SGA** ^a^					
Continuous	923	1.098 (1.061, 1.136)	<0.001	1.070 (1.025, 1.117)	0.002
Categories					
≤3	772	Reference		Reference	
>3	151	2.299 (1.490, 3.547)	<0.001	1.441 (0.899, 2.310)	0.128
**NLR** ^b^					
Continuous	923	1.246 (1.172, 1.325)	<0.001	1.199 (1.119, 1.285)	<0.001
Categories					
<3	688	Reference		Reference	
≥3	235	3.011 (2.027, 4.473)	<0.001	2.359 (1.563, 3.560)	<0.001
Quartiles					
1	228	Reference		Reference	
2	231	1.146 (0.565, 2.326)	0.705	1.286 (0.632, 2.618)	0.488
3	229	1.436 (0.735, 2.808)	0.290	1.380 (0.705, 2.701)	0.348
4	235	3.614 (1.986, 6.575)	<0.001	2.899 (1.574, 5.337)	0.001
**PG-SGA and NLR** ^c^					
Low PG-SGA and low NLR	605	Reference		Reference	
High PG-SGA and low NLR	83	0.874 (0.373, 2.044)	0.755	0.665 (0.280, 1.579)	0.355
Low PG-SGA and high NLR	167	1.951 (1.184, 3.214)	0.009	1.696 (1.022, 2.814)	0.041
High PG-SGA and high NLR	68	6.187 (3.748, 10.211)	<0.001	4.434 (2.602, 7.555)	<0.001

NPC, nasopharyngeal carcinoma; PG-SGA, Patient-Generated Subjective Global Assessment; NLR, neutrophil-to-lymphocyte ratio; OS, overall survival; HR, hazard ratio; CI, confidence interval.

a Models were adjusted for sex, age, body mass index, alcohol consumption, TNM stage, and NLR (as a continuous variable).

b Models were adjusted for sex, age, body mass index, alcohol consumption, TNM stage, and PG-SGA (as a continuous variable).

c Models were adjusted for sex, age, body mass index, alcohol consumption, and TNM stage.

**Figure 3:**
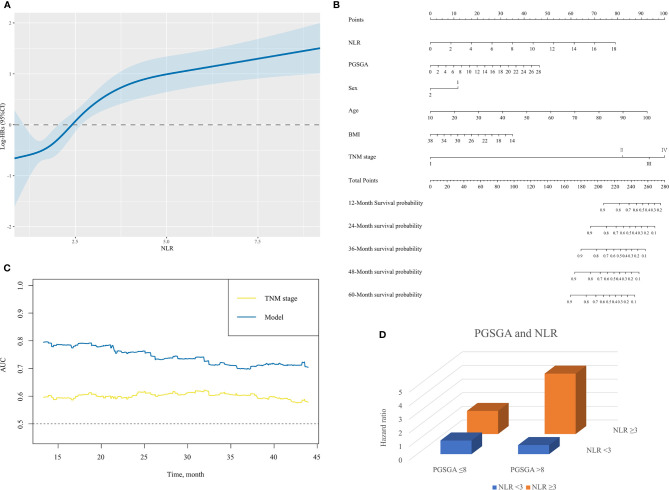
**(A)** The association between NLR (continuous) and hazard ratio of overall survival for patients with NPC undergoing chemoradiotherapy. Splines are adjusted by sex, age, body mass index, alcohol consumption, TNM stage and PG-SGA. **(B)** The nomogram for predicting the 12- to 60-month overall survival time of patients with NPC undergoing chemoradiotherapy. In the nomogram, an individual patient’s value is located on each variable axis. Based on the number of points received for each corresponding value, the sum of these numbers is located on the total point axis to determine the likelihood of 12- to 60-month survival in the matched survival axes. **(C)** A summary chart merged the AUC for prediction of survival at 12, 24, 36, or 48 months by nomogram (model) and TNM stage. The predictive capacity of model had a significant improvement as compared with TNM stage. **(D)** The interactive association of NLR and PG-SGA for patients with NPC undergoing chemoradiotherapy, presented by three-dimensional bar diagram. NLR, neutrophil-to-lymphocyte ratio; NPC, nasopharyngeal carcinoma; PG-SGA, Patient-Generated Subjective Global Assessment; AUC, area under the receiver operating characteristic curve.

When analyzed as a continuous variable, restricted cubic spline plot showed multivariable-adjusted HRs (95% CI) for NLR, which had an inverted L-shaped dose–response association with the all-cause mortality risk in NPC patients (**Figure 3A**).

### The Nomogram for Predicting the 12–60 Months’ Overall Survival Time of Patients With Nasopharyngeal Carcinoma Undergoing Chemoradiotherapy

A nomogram for the 12-, 24-, 36-, 48-, and 60-month OS based on those factors (NLR, PG-SGA, sex, age, BMI, and TNM stage) was developed for predicting OS in patients with NPC undergoing chemoradiotherapy (**Figure 3B**). Scales of the nomogram reflected coefficients from the Cox model converted to a practical 100-point range, and OS was accurately predicted by the nomogram (model) with an AUC of 0.803, 0.759, 0.700, and 0.727 for prediction of mortality in comparison with TNM stage (0.593, 0.603, 0.603, 0.591, respectively) at 12, 24, 36, and 48 months (**Figures 3C** and **Supplementary Figure S1**). The C-index for OS prediction of nomogram was 0.75 (95% CI, 0.70–0.80), better than that of TNM stage 0.60 (0.54–0.65).

### Interactive Association of Exposures With Mortality Between Patient-Generated Subjective Global Assessment and Neutrophil-to-Lymphocyte Ratio

**Table 3** and **Figure 3D** illustrate the HR for each condition of exposure combinations compared with the referent: low PG-SGA (PG-SGA score ≤3) and low NLR (NLR < 3) group. The interaction of PG-SGA with NLR was significant (p = 0.009). Patients in NPC with high PG-SGA and high NLR had a nearly 4.5-fold increased risk of death (HR = 4.43, 95% CI = 2.60–7.56) as compared with patients with low PG-SGA and low NLR. PG-SGA and NLR showed synergism with each other.

## Discussion

This was a large multicenter study comprising 923 patients with NPC undergoing chemoradiotherapy across several provinces in China, which investigated the relationships between the nutritional status and patients’ prognosis through a long-term follow-up. Malnutrition is commonly seen in patients with NPC undergoing chemoradiotherapy (9, 16, 22). Patients with NPC suffer from malnutrition caused by complex factors, including the following: 1) the tumor invades the basis cranii, causing paralysis of the glossopharyngeal nerve, which leads to dysphagia (23). 2) As a consequence of injuries to normal tissues, such as the oral mucosa, taste buds, and salivary glands, radiation-induced oral mucositis and other complications are very common in patients with NPC undergoing RT, causing difficulty in chewing and swallowing (24, 25). 3) The concurrent chemotherapy often cause severe side effects of gastrointestinal tract such as asitia, nausea, and vomiting, which lead to decreased appetite and gastrointestinal dysfunction (26). 4) Patients with NPC also suffer from emotional disorders such as fear, anxiety, and depression, which further impair the patient’s ability to eat and digestive function (19, 27). 5) The insufficient nutritional supply or unreasonable ingredients was found in the nursing care of patients with NPC, due to patients’ and their relatives’ poor nutritional knowledge. However, few data are available regarding the nutritional status of patients with NPC undergoing chemoradiotherapy. This study investigated the nutritional status of patients with NPC who received RT, chemotherapy, or both, on the basis of PG-SGA score and BMI. In addition, an NLR was established to reflect patients’ systemic inflammation (28), and the correlation between PG-SGA and NLR on survival time was explored.

Based on our results, we found that PG-SGA score and NLR at admission were associated with poor prognosis of NPC undergoing chemoradiotherapy (**Figures 2A**–**C** and **Tables 2**, **3**). NLR had an inverted L-shape relationship with mortality risk among patients with NPC undergoing chemoradiotherapy (**Figure 3A**). Furthermore, we established a nomogram model based on domains of NLR, PG-SGA, sex, age, BMI, and tumor stage to predict the OS rate associated with NPC patients undergoing chemoradiotherapy. The nomogram was able to predict an individual’s risk of 12-, 24-, 36-, 48-, and 60-month OS rates with relatively good accuracy, especially for predicting the 12-month OS rates (**Figures 3B, C** and **Supplementary Figure S1**). Finally, the co-occurrence of high NLR and high PG-SGA score was associated with a nearly 4.5-fold increased risk of mortality among patients with NPC undergoing chemoradiotherapy (**Table 3** and **Figure 3D**). To sum up, there was a potential synergistic effect of systemic inflammation and severe malnutrition with mortality risks for patients with NPC who received RT, chemotherapy, or both.

In recent years, an increasing number of studies have demonstrated that measures of inflammation and malnutrition are each independently powerful prognostic indicator in patients with cancer (28). A prospective cohort (17) of 2,470 patients with stage I to III CRC revealed that pre-diagnosis inflammation was associated with at-diagnosis sarcopenia. OS was worse in the group with simultaneous existence of sarcopenia and high NLR, which seem to be consistent with our results. In previous studies (18), the level of NLR in patients with cancer cachexia is generally higher than that in patients without cancer cachexia, and the high NLR level was related to poorer survival of cancer patients, indicating that NLR could be used as an independent prognostic factor for patients with cachexia. Wang et al. reported that in patients with NPC, weight loss makes them more vulnerable to recurrent pneumonia. Notably, our results suggest likely synergistic effects between inflammation and malnutrition for patients with NPC undergoing chemoradiotherapy, which explains why nutritional therapy for patients with NPC could be beneficial to reduce systemic inflammation and improve the immunity of patients.

The close connections of nutrition, inflammation, and cancer have been attracting our attentions for many years. Our findings also indicated the effect of chemoradiotherapy on the nutritional status of patients with NPC. In addition to malnutrition, chemoradiotherapy may induce mucositis, tissue damage, and acute toxicities, which may cause systemic inflammatory response (9, 29). Thus, malnutrition and systemic inflammation may have a prognostic role in the process of chemoradiotherapy. Malnutrition expended massive protein, which further disturbs metabolic function, impairs immunity, increases susceptibility to infection, compromises the response to antitumor treatments, and aggravates chemoradiotherapy toxicity, hence severely affecting survival and outcomes (4, 22, 25, 30). Additionally, protein–energy malnutrition may induce a high level of systemic inflammatory factors, such as tumor necrosis factor (TNF)-α and interleukin-6, can facilitate tumor cell proliferation invasion and metastasis, and can enhance malignant properties (31). As a consequence, malnutrition was also an indicator of a systemic inflammatory response. In turn, pro-inflammatory cytokines and tumor factors are involved in high catabolism in the inflammatory state, which consumes a large amount of the patient’s skeletal muscle mass and caloric intake (17). This metabolic disorder will eventually lead to continuous weight loss and deterioration of nutritional status, damage to tissue and organ function, increase in the incidence of complications, and decrease in survival time. More importantly, inflammation plays an important role in the occurrence and progression of malignant tumors. Malnutrition, inflammation, and cancer really have become a vicious circle. Therefore, it is particularly important for cancer patients with malnutrition to control systemic inflammatory; and for cancer patients with high levels of inflammation, it is necessary to pay more attention to early nutritional intervention to better improve the prognosis of cancer patients. Interestingly, now that the survival rates of patients with high NLR may potentially benefit from nutritional therapy in our results, some special nutritional support with anti-inflammatory function should be given more to the patients with NPC undergoing chemoradiotherapy, such as ω-3 polyunsaturated fatty acid (ω-3 PUFA)-enriched and β-hydroxy-β-methylbutyrate (HMB)-enriched supplement. ω-3 PUFA has already been widely recognized as one of the anti-inflammatory nutrients (32). HMB, an intermediate metabolite of leucine that exists in human muscle cells, has been shown to inhibit protein degradation, attenuate the decrease in protein synthesis, and improve nutritional status (33). Moreover, one study (34) found that HMB attenuated the inflammatory effect of lipopolysaccharide (LPS), TNF, interferon (IFN), and angiotensin II (ANG II). A randomized trial (35) examined the effects of HMB supplementation on inflammation, protein metabolism, and pulmonary function in patients with chronic obstructive pulmonary disease, requiring mechanical ventilation. After 7 days of intervention, patients who were treated with HMB showed consistent reductions in CRP and in white blood cell counts from baseline, suggesting that HMB plays a positive role in the treatment of chronic inflammation.

To our knowledge, this is one of the meaningful studies on NPC to examine the relationship between malnutrition and biomarkers of systemic inflammation and the only study to examine whether high NLR and high PG-SGA score have a synergetic interaction on the NPC patient’s survival risk. The survival difference among the four groups (different combinations of PG-SGA score and NLR) indicates that prolonged survival needs not only a better nutrition status but also low-level systemic inflammation, which is a significant issue that should not be underestimated. Similar to all clinical research, our study was subject to several limitations: first, we could not examine other markers of systemic inflammation, such as PNI (19), PLR (20), and CRP levels (21). Because the related data are so insufficient or inappropriate, we had to stop analyzing them. Second, we did not study whether dynamic NLR could modify the prognostic value. Moreover, other covariates were collected using electronic medical record, questionnaire, or a one-on-one interview; and errors in measurement were inevitable. Finally, different diagnostic and therapeutic levels in the various hospitals might have masked the true associations. Further data are required to confirm these findings and to increase the precision of the effect for these exposures.

## Conclusions

Malnutrition and systemic inflammation are common in patients with NPC. The PG-SGA score and NLR reflect the integrated nutrition status and inflammatory level of NPC patients, respectively. Our study provided clear evidence that high PG-SGA score and high NLR adversely affect the OS of patients with NPC undergoing chemoradiotherapy, suggesting that PG-SGA score and NLR were independent prognostic indicators for patients with NPC undergoing chemoradiotherapy. We also found that PG-SGA and NLR were interactive with each other. The co-occurrence of malnutrition and systemic inflammation at admission identified patients with a nearly 4.5-fold increased risk of mortality as compared with patients with neither condition. Therefore, on the one hand, it is particularly important for cancer patients with malnutrition to control systemic inflammation; on the other hand, for cancer patients with high levels of inflammation, it is necessary to offer nutritional intervention as early as possible to better improve the prognosis of cancer patients. Future studies are required to dynamically explore specific relationships and exact mechanisms between nutrition status and systemic inflammation and could assist to develop better strategies to increase the survival time and QoL of patients with NPC undergoing chemoradiotherapy.

## Data Availability Statement

The raw data supporting the conclusions of this article will be made available by the authors, without undue reservation.

## Ethics Statement

Written informed consent was obtained from the individual(s) for the publication of any potentially identifiable images or data included in this article.

## Author Contributions

HS and XW are responsible for the study design, conceptualization, systematic review, project administration, the decision to publish, and manuscript preparation. MY, YG, and MT helped conduct the statistical analyses. BR and YC revised and edited the manuscript. ZG, HX, and MC provided data support from INSCOC project. All authors contributed to the article and approved the submitted version.

## Funding

This study was supported by a National Science and Technology Major Project from Ministry of Science and Technology of the People’s Republic of China, the National Key Research and Development Program (No: 2017YFC1309200; Title: The key technology of palliative care and nursing for cancer patients).

## Conflict of Interest

The authors declare that the research was conducted in the absence of any commercial or financial relationships that could be construed as a potential conflict of interest.

## Publisher’s Note

All claims expressed in this article are solely those of the authors and do not necessarily represent those of their affiliated organizations, or those of the publisher, the editors and the reviewers. Any product that may be evaluated in this article, or claim that may be made by its manufacturer, is not guaranteed or endorsed by the publisher.

## Supplementary Material

Supplementary Figure 1 In ROC curve analysis, comparisons of the AUC for prediction of survival at 12- or 24- or 36- or 48- month by nomogram and TNM stage. NPC, Nasopharyngeal carcinoma; PGSGA, Patient-Generated Subjective Global Assessment; NLR, neutrocyte to lymphocyte ratio; HR, hazard ratio; CI, confidence interval; BMI, body mass index; ROC, receiver operator characteristic; AUC, area under curve.
