# Supplementary figures and images for: Association of Systemic Inflammation and Malnutrition With Survival in Nasopharyngeal Carcinoma Undergoing Chemoradiotherapy: Results From a Multicenter Cohort Study
Source: Front Oncol. 2021 Oct 26;11:766398. doi: 10.3389/fonc.2021.766398 (PMC8576523; doi:10.3389/fonc.2021.766398)

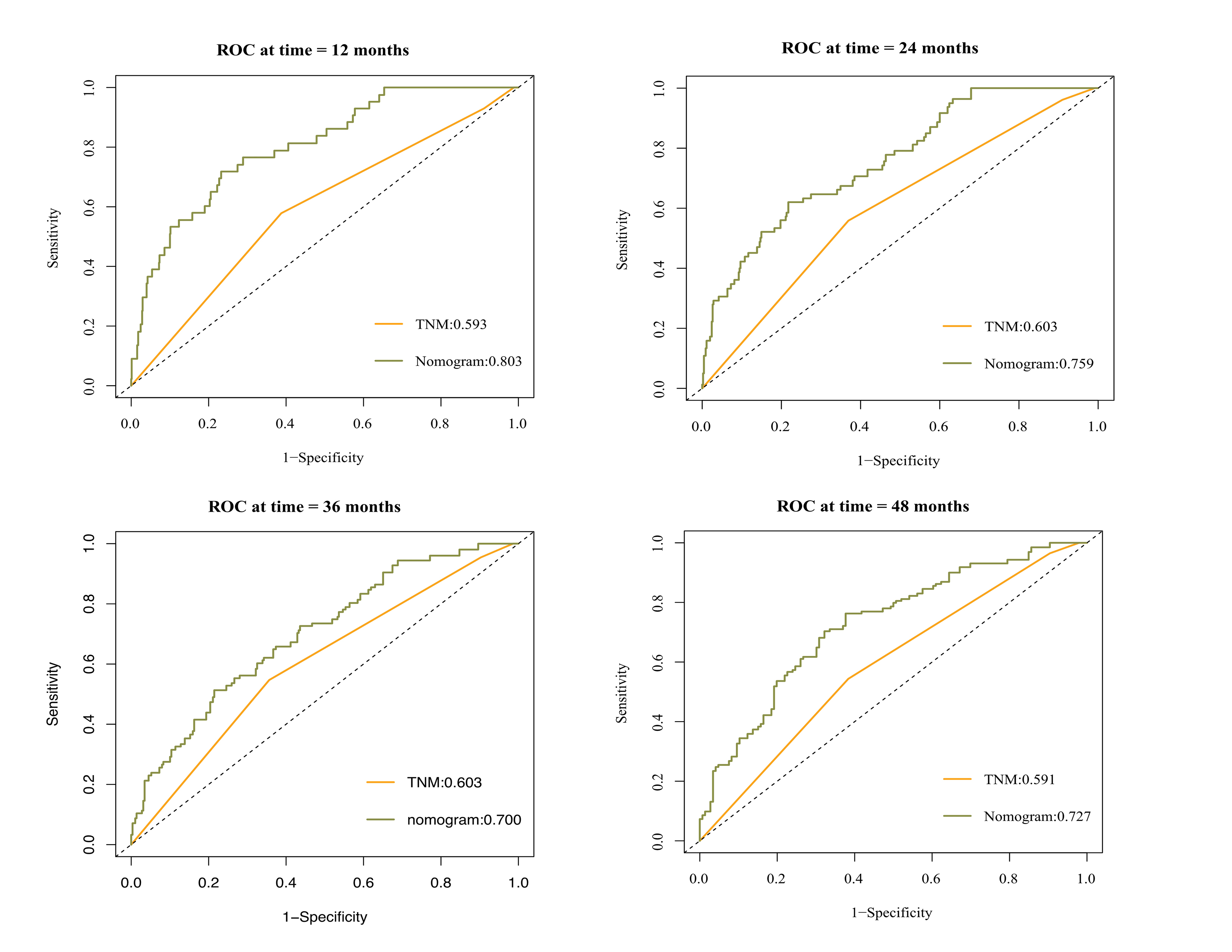

Supplement: Supplementary Figure 1 — In ROC curve analysis, comparisons of the AUC for prediction of survival at 12- or 24- or 36- or 48- month by nomogram and TNM stage. NPC, Nasopharyngeal carcinoma; PGSGA, Patient-Generated Subjective Global Assessment; NLR, neutrocyte to lymphocyte ratio; HR, hazard ratio; CI, confidence interval; BMI, body mass index; ROC, receiver operator characteristic; AUC, area under curve. [file Image_1.jpeg]
